# The View Outside of the Box: Reporting Outcomes Following Radical Cystectomy Using Pentafecta From a Multicenter Retrospective Analysis

**DOI:** 10.3389/fonc.2022.841852

**Published:** 2022-01-26

**Authors:** Łukasz Zapała, Aleksander Ślusarczyk, Bartłomiej Korczak, Paweł Kurzyna, Mikołaj Leki, Piotr Lipiński, Jerzy Miłow, Michał Niemczyk, Kamil Pocheć, Michał Późniak, Maciej Przudzik, Tomasz Suchojad, Rafał Wolański, Piotr Zapała, Tomasz Drewa, Marek Roslan, Waldemar Różański, Andrzej Wróbel, Piotr Radziszewski

**Affiliations:** ^1^ Clinic of General, Oncological and Functional Urology, Medical University of Warsaw, Warsaw, Poland; ^2^ Department of Urology, Regional Specialist Hospital, Kielce, Poland; ^3^ 2nd Clinic of Urology, Medical University of Lodz, Łódź, Poland; ^4^ Clinic of Urology, University Hospital No. 1, Bydgoszcz, Poland; ^5^ Department of Urology, Faculty of Medicine, University of Warmia and Mazury, Olsztyn, Poland; ^6^ 2nd Clinic of Gynecology, Medical University of Lublin, Lublin, Poland

**Keywords:** radical cystectomy, quality of cystectomy, pentafecta, bladder cancer, muscle-invasive bladder cancer

## Abstract

We aimed at characterization of the patients undergoing radical cystectomy (RC) using the prognostic model (a modified pentafecta). In the multicenter retrospective study, we enrolled 304 patients with bladder cancer (pTis-4N0-2M0) who underwent RC between 2015 and 2020 in experienced centers. The definition of the pentafecta was as follows: no Clavien–Dindo grade III–V complications at 90 days and no long-term complications related to urinary diversion <12 months, negative surgical margins, ≥10 lymph nodes (LNs) resected, and no recurrence ≤12 months. RC-pentafecta achievement rate was 22% (n = 67), varying from 47% to 88% attainment rate for different pentafecta components, and was the lowest for sufficient LN yield. Both 12-month recurrence-free survival (RFS) and cancer-specific mortality were compromised in pentafecta failers compared with achievers (57.8% vs. 100% and 33.8% vs. 1.5%, respectively). The following were identified as crucial predictors of RC pentafecta achievement: modality of the surgery, type of urinary diversion, histological type of bladder cancer, advanced staging, and elevated preoperative serum creatinine. In conclusion, we found that the pentafecta achievement rate was low even in high-volume centers in patients undergoing cystectomy. The complexity of the procedure directly influenced the attainment rate, which in turn led to an increase in cancer-specific mortality rate among the pentafecta failers.

## Introduction

The worldwide results of the treatment of muscle-invasive bladder cancer (MIBC) bring the image of suboptimal management of the disease with 50% of patients undergoing radical cystectomy (RC) not surviving 5 years (1). The majority of authors focus on the oncological reasons that impose low survival rates in this group of patients on the basis of widely approved risk factors and suggest performing a cystectomy at earlier stages (2). Scarce data exist concerning the possible connection with the quality of the surgery and performance of the centers, though (3). The main issues mentioned in the recent papers are the number of cases operated annually as the predictor of the effectiveness (4).

Predominantly, RC is performed in elderly patients with advanced cancer burden (5). According to various authors, it requires a certain amount of time for the procedure itself (4–7 h) and hospitalization (4–17 days) (6–10). Due to the duration and complexity of the surgery, as well as the general condition and age of the individuals qualified for RC, the vast majority of patients experience early postoperative complications, and the mortality rate in some studies reaches 10% within 90 days since surgery (6, 10). Even two-thirds of patients operated on in the world experience at least one complication within the 90-day follow-up period (6, 11, 12), while approximately 60% of complications are linked to the implementation of specific urinary diversion (UD) (13). Moreover, it has been shown that conducting the procedure by an experienced surgeon (a significant number of cases performed per year) and the experience of a center, in which a sufficient number of RCs are performed annually, allows to reduce the risk of complications and to limit the perioperative mortality (5, 7, 8, 14). The idea of including a learning curve as a factor influencing the quality of the surgery is crucial especially in truly sophisticated RC, i.e., robot-assisted with intracorporeal diversion (15), even though it is characterized by the equivalent oncologic outcomes as open RC in intermediate-term (1, 2, 3, and 4 years after surgery) survival analysis (16). Most authors define the minimum number of operations as 30 conducted each year (so-called high-volume center), but recent series suggest that the performance of at least 10 cystectomies per year leads to the significant reduction of both the 30- and 90-day mortality (5). Thus, single surgeon analysis may further facilitate the selection of the clinical factors and help to validate tools based on them, e.g., the Quality Cystectomy Score (QCS) to measure the surgical performance and assist the introduction of novel techniques (17).

There were standardized outcome criteria proposed for reporting purposes, i.e., trifecta and/or pentafecta for partial nephrectomy and radical prostatectomy, which focused on both the quality of cancer management and the assessment of surgical complications (18, 19). It seems reasonable to promote the idea of incorporating similar scales in the cohort of cystectomized patients in the light of growing interest in the technical quality of the procedure and the changing landscape of open surgery indications (20–23). RC pentafecta assessment was designed to serve as a standardized tool for the measurement of surgical quality, which directly influences oncological and functional outcomes. Even though different definitions and modifications of these models are available, the one proposed by Aziz et al. (20) and revised by Cacciamani et al. (21) are most commonly cited. The debate, however, continues about the value and number of resected lymph nodes (LNs), and many authors lower the threshold to 10 and 15 ones (24–26). It is also speculated about other possible explanations of incoherent data on the influence of lymphadenectomy on survival, e.g., due to the technique of nodal submission (separate templates) or pathological reporting standards (27).

Here, we reintroduced the idea of the assessment model to be used for the quality analysis of RC. We aimed at characterization of the patients’ population undergoing RC, with particular emphasis on perioperative complications and the effectiveness of oncological treatment along with survival data, using the prognostic model that was constructed from clinical and histopathological variables (a modified pentafecta assessment). The tool was then used to evaluate the quality of RC treatment based on the attainment of the aforementioned pentafecta. Thus, the primary endpoint was to analyze the RC pentafecta in the present cohort, while the second was to determine the influence of attaining pentafecta on oncological outcomes and to define the risk factors compromising the pentafecta achievement.

## Materials and Methods

### Study Group

In the present retrospective study, five oncological centers with established experience meet the abovementioned criteria (performance of at least 10 RCs annually), including three high-volume centers (over 30 cystectomies yearly) (5). The analysis comprised consecutive patients with bladder cancer staged pTis-4N0-2M0 who were qualified according to the European Association of Urology (EAU) Guidelines (5) and underwent standard RC (open or laparoscopic) with pelvic lymphadenectomy (extended if ≥10 nodes) in four tertiary centers and one non-tertiary center between 2015 and 2020. The decision about the type of UD (performed extracorporeally) was made considering patient- and disease-associated factors or surgeon preference. Systematic preoperative workup included the following: routine laboratory tests, contrast-enhanced CT imaging of the chest, abdomen, and pelvis and pathological report available from the initial transurethral resection of bladder tumor.

The following data were collected: gender, age, American Association of Anesthesiology (ASA) score, body mass index (BMI) score, smoking status, neoadjuvant systemic treatment, pre- and postoperative disease staging using the TNM scale based on imaging studies and pathological reports (28), duration of surgery, surgical modality, type of UD (orthotopic neobladder; ileal conduit; uretero-ureterocutaneostomy (UCS); transuretero-ureterocutaneostomy (TU-UCS)), the incidence of positive surgical margin (PSM; defined as both urethral/ureteral and perivesical soft tissues), number of resected LNs, length of hospitalization (LOH), rehospitalization status, histopathological report after surgery, the occurrence of early/late complications classified according to the Clavien–Dindo scale (29), and separate blood transfusions, with hemoglobin and creatinine concentrations before and after surgery. All data were obtained from the medical charts and databases from the enrolled centers. Additionally, telemedicine visits were conducted considering incomplete follow-up details, whenever necessary.

Firstly, Aziz et al. developed an RC-pentafecta idea based on expert opinions (20). Cacciamani et al. modified this RC-pentafecta, incorporating two measures of perioperative morbidity and including three measures of oncological effectiveness (21). During the analysis of the results of treatment of patients undergoing RC, the following definition of pentafecta was used, modified after Cacciamani et al. (21):

- no Clavien–Dindo grade III–V complications at 90 days and no long-term complications related to UD < 12 months

- negative surgical margins (all soft tissue and urethral and ureteral) in post-cystectomy pathological report, ≥10 LN removal during the procedure, and no clinical relapse of bladder cancer ≤12 months after RC found in the follow-up CT. Based on other authors’ observations, we included the threshold of 10 LNs in further analyses (24–26).

The following definitions were incorporated for the purpose of the present analyses: early complications, occurring within 90 days post cystectomy; late complications, occurring over 90 days but no more than 12 months after cystectomy; and LOH, all the periods of hospital stay (30). Overall survival (OS) was defined as the time from cystectomy to death from any cause, while cancer-specific survival (CSS) was defined as time to death related to bladder cancer, excluding 30-day perioperative mortality cases.

All patients signed an informed consent form, and the study was conducted under the ethics committee vote AKBE/133/2021 of the Medical University of Warsaw.

### Statistical Analyses

All statistical analyses were performed in SAS software version 9.4. Results are presented in respective tables as the number of patients and percentage for categorical variables and medians, accompanied by the interquartile range (IQR) for continuous variables. Fisher’s test or chi-square test for categorized variables and Mann–Whitney U test for continuous variables were used to determine the differences between groups. The differences in the time to death were analyzed using the Kaplan–Meier method and log-rank test. In univariable analyses, logistic regression was utilized to identify factors associated with respective outcomes (e.g., pentafecta achievement). Relevant factors from univariate analyses were used for further multivariable analysis. Odds ratio (OR) supplemented with 95% CI were derived from logistic regression. For all statistical analyses, we considered a two-sided p-value <0.05 as statistically significant.

## Results

### Basic Characteristic of the Cohort

Three hundred four patients who underwent RC were included in the study. Standard neoadjuvant chemotherapy (NAC) was implemented in 76 cases (25%). The median age of the patients in the studied cohort was 68 years, and most patients were male (75%). Over 81% of patients were treated with cystectomy because of MIBC (cT2–T4), and in the vast majority of cases, the final pathological results proved the urothelial histology of cancer. Median postoperative follow-up was 18.5 months (IQR 11.6–33 months). Baseline characteristics of the cohort and subgroups stratified according to the RC-pentafecta achievement are presented in **Table 1**.

**Table 1:** Baseline characteristics of patients who underwent RCs.

			RC-pentafecta achievement				
	Total		No		Yes		
	No./mean	%/IQR	No./mean	%/IQR	No./mean	%/IQR	
Age	68	63–72	68	64–72	66	60–72	0.24
BMI (kg/m^2^)	25.8	22–29	25.3	22–29	26.5	24–29.5	0.19
Hemoglobin (g/dl)	12.5	10.8–13.8	12.4	10.8–13.8	13	10.6–14.3	0.36
Creatinine (mg/dl)	1.06	0.85–1.35	1.1	0.88–1.1	0.88	0.81–1.16	0.0005
Male	228	75	177	74.68	51	76.12	0.87
Hematuria	178	58.55	124	55.61	54	83.08	<0.0001
Hydronephrosis	106	34.87	88	37.13	18	26.87	0.15
ASA score							
1	16	5.26	14	5.91	2	2.99	0.067
2	125	41.12	98	41.35	27	40.30	
3	147	48.36	109	45.99	38	56.72	
4	16	5.26	16	6.75	0	0.00	
Smoking status							
Active	97	36.06	80	38.83	17	26.98	0.17
Former	115	42.75	82	39.81	33	52.38	
Never	57	21.19	44	21.36	13	20.63	
Neoadjuvant chemotherapy	76	25.0	62	26.16	14	20.90	0.42
Preoperative cT							
cT0	4	1.32	2	0.84	2	2.99	0.72
cT1	45	14.8	34	14.35	11	16.42	
cT2	158	51.97	125	52.74	33	49.25	
cT3	59	19.41	45	18.99	14	20.90	
cT4	31	10.2	24	10.13	7	10.45	
CIS	5	1.64	5	2.11	0	0.00	
Ta	2	0.66	2	0.84	0	0	
Preoperative cN							
cN0	200	65.79	143	60.34	57	85.07	<0.0001
cN1	15	4.93	10	4.22	5	7.46	
cN2	19	6.25	17	7.17	2	2.99	
cN3	10	3.29	9	3.80	1	1.49	
cNx	60	19.74	58	24.47	2	2.99	

RC, radical cystectomy; IQR, interquartile range; ASA, American Association of Anesthesiology; CIS, carcinoma in situ.

### Pentafecta Details

RC-pentafecta achievement rate was 22%. The following components included in the RC-pentafecta assessment were achieved in the lowest percentage: 12-month recurrence-free survival (RFS) of 64.8%, and ≥10 LNs in the RC specimen of 47% (**Table 2**).

**Table 2:** Summary of pentafecta criteria achievement.

Pentafecta components met	No. of pts	%
12-month RFS	197	64.8
Resection of ≥10 lymph nodes	143	47.0
Negative surgical margin	266	87.5
Absence of major complications	57	81.3
Absence of UD-related complications	268	88.2
Overall pentafecta achievement	67	22%

RFS, recurrence-free survival; UD, urinary diversion.

Patients, who attained pentafecta, less frequently underwent open vs. laparoscopic surgery (54% vs. 94%) and more frequently received ileal conduit (64% vs. 38%) or orthotopic neobladder (9% vs. 0.8%), or UD, when compared with patients who did not attain pentafecta. In general, readmissions were more frequent in pentafecta failers (40.5% vs. 21%), but no significant difference was observed for early rehospitalizations 90 days) (25% vs. 18%). For details regarding the surgery and perioperative complications, please refer to **Table 3**.

**Table 3:** Radical cystectomy details and perioperative outcomes in patients who achieved or not pentafecta.

			RC-pentafecta achievement				
	Total		No		Yes		
	No./mean	%/IQR	No./mean	%/IQR	No./mean	%/IQR	
Modality of surgery							
Laparoscopy	45	14.8	14	5.91	31	46.27	<0.0001
Open	259	85.2	223	94.09	36	53.73	
Urinary diversion type							<0.0001
Ileal conduit	134	42.43	91	38.4	43	64.18	
Orthotopic neobladder	8	2.63	2	0.84	6	8.96	
UCS	136	44.74	129	54.43	7	10.45	
TU-UCS	24	7.89	13	5.49	11	16.42	
Other	2	0.99	2	0.84	0	0	
LOH (days)	13	9–17	14	9–19	11	8–15	0.001
Clavien–Dindo^*^							<0.0001
1	127	41.78	85	35.86	42	62.69	
2	120	39.47	95	40.08	25	37.31	
3	43	14.14	43	18.14	0	0.00	
4	7	2.3	7	2.95	0	0.00	
5	7	2.3	7	2.95	0	0.00	
Blood transfusion	143	47.04	124	52.32	19	28.36	0.0005
Readmission < 90 days	71	23.36	59	24.89	12	17.91	0.26
Readmission	110	36.18	96	40.51	14	20.90	0.004
UD-related complications^*^	36	11.84	36	15.19	0	0.00	0.0001
LN count removed	9	4–17	7	3–12	20	13–31	<0.0001

LN, lymph nodes; UD, urinary diversion; RC, radical cystectomy; IQR, interquartile range; UCS, uretero-ureterocutaneostomy; TU-UCS, transuretero-ureterocutaneostomy; LOH, length of hospitalization.

^*^Pentafecta variables.

### Oncological Results and Survival Analysis With Respect to the Achievement of Pentafecta

In patients who did not attain RC-pentafecta, the pathological T staging of bladder cancer was more advanced (more often pT3–T4 tumors), the nodal staging was greater, and non-pure urothelial histological type of cancer was found more frequently. The preoperative staging regarding the LN assessment differed between groups of RC-pentafecta achievers and failures. Only 60% of pentafecta failures had cN0 compared with 85% of achievers (p < 0.0001) (**Table 1**). Postoperatively, 63.7% of former ones and 82% of latter ones were assessed as pN0 stage in a pathological specimen (p = 0.007) (**Table 4**). Estimated 3-year OS in the whole cohort reached 42%, while estimated CSS was 48% at 3 years after RC. The 12-month RFS was significantly compromised in patients not achieving pentafecta (57.8% vs. 100%). Cancer-specific mortality was dramatically higher in RC-pentafecta failers compared with achievers (33.8% vs. 1.5%). Oncological results are presented in **Table 4**.

**Table 4:** Oncological outcomes of patients who underwent RC and achieved or not pentafecta.

			RC-pentafecta achievement				
	Total		No		Yes		
	No./mean	%/IQR	No./mean	%/IQR	No./mean	%/IQR	
Staging T							0.001
pT0	22	7.24	17	7.17	5	7.46	
pT1	36	11.84	23	9.70	13	19.40	
pT2	85	27.96	61	25.74	24	35.82	
pT3	80	26.32	67	28.27	13	19.40	
pT4	54	17.76	52	21.94	2	2.99	
CIS	19	6.25	12	5.06	7	10.45	
Ta	8	2.63	5	2.11	3	4.48	
MIBC	219	72.04	180	75.95	39	58.21	0.006
pT3–4	134	44.08	119	50.21	15	22.39	<0.0001
pN							
pN0	206	67.76	151	63.71	55	82.09	0.007
pN1	42	13.82	35	14.77	7	10.45	
pN2	25	8.22	22	9.28	3	4.48	
pN3	7	2.3	5	2.11	2	2.99	
pNx	24	7.89	24	10.13	0	0.00	
Histology type							
Pure urothelial	263	86.51	211	89.03	52	77.61	0.024
Other	41	13.49	26	10.97	15	22.39	
Pentafecta components							
12-month RFS^*^	197	64.8	130	57.78	67	100.00	<0.0001
LND ≥ 10^*^	143	47.04	76	32.07	67	100.00	<0.0001
Negative SM^*^	266	87.5	199	83.97	67	100.00	<0.0001
Oncological outcomes							
Recurrence	106	34.87	105	45.85	1	1.49	<0.0001
Overall mortality	105	34.54	103	43.64	2	2.99	<0.0001
Cancer-specific mortality	81	26.64	80	33.76	1	1.49	<0.0001

RC, radical cystectomy; IQR, interquartile range; CIS, carcinoma in situ; MIBC, muscle-invasive bladder cancer; RFS, recurrence-free survival; LND, lymph node density.

^*^Pentafecta variables. SM, surgical margin.

**Figure 1:**
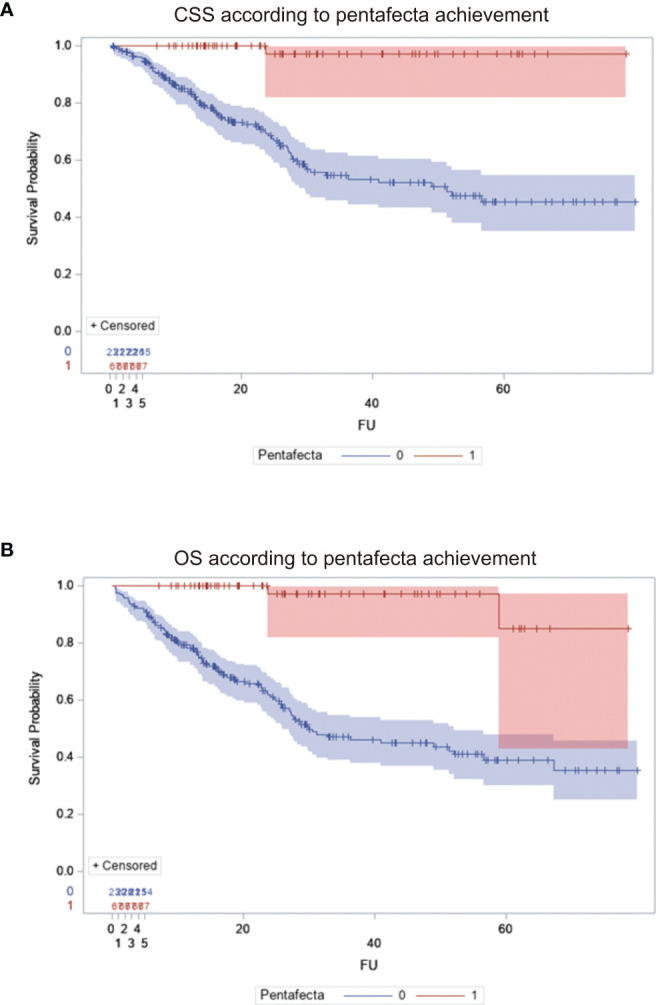
Survival of patients undergoing radical cystectomy: comparison between patients who achieved and those who failed to achieve RC-pentafecta. **(A)** Cancer-specific survival. **(B)** Overall survival. Blue line, pentafecta failers; red line, pentafecta achievers.

Differences in survival between the abovementioned groups were demonstrated using the Kaplan–Meier curves (**Figure 1**).

### Univariate and Multivariate Analyses of Pentafecta Achievement

The following factors were identified as crucial predictors of RC pentafecta achievement: modality of the surgery, type of UD, pure urothelial histology of bladder cancer, advanced staging (pT3–T4), and elevated preoperative serum creatinine >1 mg/dl. For detailed univariate and multivariate analyses, please see **Table 5**.

**Table 5:** Factors influencing pentafecta achievement: univariate (A) and multivariate analysis (B).

A	OR	95% CI	p-Value	
Age	0.988	0.956–1.020	0.4601	
Male gender	1.081	0.574–2.036	0.8106	
Ever smoker	1.044	0.521–2.093	0.9027	
Hydronephrosis	0.595	0.315–1.121	0.1082	
NAC	0.746	0.387–1.437	0.3807	
Open surgery	0.422	0.242–0.737	0.0024	
BMI	1.031	0.969–1.097	0.3299	
Urinary diversion			<0.0001	
UCS/TU-UCS	Ref			
Ileal conduit	3.728	2.026–6.860		
Orthotopic neobladder	23.667	4.439–126.194		
Other diversions	–	0.001–999		
MIBC	0.441	0.250–0.780	0.0049	
pT3–T4	0.286	0.153–0.536	<0.0001	
Pure urothelial histology	0.427	0.211–0.864	0.0179	
Transfusion	0.361	0.200–0.650	0.0007	
Preop. Hemoglobin	1.042	0.912–1.190	0.5459	
Creatinine > 1 mg/dl	0.344	0.195–0.606	0.0002	
**B**				
Open surgery	0.349	0.172–0.707	<0.0001	
Urinary diversion				
UCS/TU-UCS	Ref			<0.0001
Ileal conduit	4.633	2.259–9.502	
Orthotopic neobladder	20.828	3.201–135.524	
Other UD	<0.001	<0.001–>999.999	
Pure urothelial histology	0.387	0.172–0.869	0.0192	
pT3–T4	0.403	0.200–0.812	0.0176	
Creatinine > 1 mg/dl	0.476	0.254–0.890	0.0062	

OR, odds ratio; NAC, neoadjuvant chemotherapy; BMI, body mass index; UCS, uretero-ureterocutaneostomy; TU-UCS, transuretero-ureterocutaneostomy; MIBC, muscle-invasive bladder cancer; UD, urinary diversion.

### Prediction of Cancer Recurrence and Major Complications After Radical Cystectomy

In multivariate analysis, factors associated independently with cancer recurrence and major complications after RC were identified and shown in **Tables 6** and **7**, respectively. Advanced staging (pT3–T4), PSMs, blood transfusion during hospitalization, lower LN counts resected, and positive LN cancer status were the factors that increased the risk of cancer recurrence after RC. The risk of recurrence is higher among patients with positive LNs (pN1–3). The risk of major complications after RC (Clavien–Dindo ≥3) was higher in patients who were obese and had elevated preoperative serum creatinine >1 mg/dl.

**Table 6:** Factors predicting cancer recurrence after RC—multivariate analysis.

	OR	95% CI	p-Value
pT3–T4	3.784	1.971–7.263	<0.0001
Negative margin	0.406	0.163–1.010	0.0495
Transfusion	1.882	1.002–3.538	0.0270
LN count	0.923	0.883–0.965	<0.0001
Lymph node status			
pN1–3	3.154	1.524–6.525	0.0005
pNx	4.271	1.325–13.763	

RC, radical cystectomy; LN, lymph node.

**Table 7:** Risk factors for major complications (Clavien–Dindo ≥3) after RC—multivariate analysis..

	OR	95% CI	p-Value
Obesity	3.061	1.474–6.355	0.0050
Creatinine > 1 mg/dl	2.146	1.057–4.357	0.0141

RC, radical cystectomy.

## Discussion

There is a growing need to develop and validate an adequate perioperative risk calculator for RC in terms of both survival and morbidity (31). Based on the recent findings, one suggests referral of patients to the selected centers, in which RC should be performed to improve the quality and outcomes following surgery (4). The presented concept of pentafecta may be useful not only for the clinicians and patients but also for reimbursement purposes and the assessment of the center by a public payer. Although regionalization of bladder cancer surgeries remains a suboptimal solution due to, e.g., overload of cancer centers, the quality assessment using pentafecta may become an additional tool to improve quality of care and operative morbidity (32). Taking into consideration the other approaches, one should remember about their poor accuracy; e.g., Mannas et al. revealed the discordance between the National Surgical Quality Improvement Program prediction rate and Clavien–Dindo observation of any serious complications (33). Therefore, a concept of tri- and pentafecta models was proposed by Aziz et al. based on the experts’ panel, members of which ranked respective variables, of which five are the most commonly selected formed pentafecta criteria (20). The idea was later developed and modified by Cacciamiani et al. (21), and other researchers analyzed it in various settings, e.g., in either open (34) or robotic approach (22, 23), while mainly single-center data are available except for single papers (22, 23).

In the current analysis of the quality of RC based on predefined factors, we found that only 22% of patients achieved the pentafecta, while some of its components, i.e., oncological and functional variables, were present in 47%–88.2% of cases. This reflects the heterogeneity of the aspects that determine the real value of the surgery and may play an important role in patients’ survival. The top influencers of the pentafecta, though, seem to be the number of resected LN and 12-month RFS, as the rest of the factors were attained in over 80% of cases. Similar findings were reported by Laymon et al.: only 33.6% of individuals who underwent open RC attained pentafecta, while the main reason for not achieving pentafecta was a low number of resected LNs (i.e., <16) (34). Another corresponding paper with our study by Baron et al. revealed that pentafecta protocol in the cohort of patients undergoing robot-assisted RC with intracorporeal UD was highly influenced by the number of resected LNs (56% of pentafecta attainment) leading to an overall pentafecta rate as of 39% (22).

Standardization may be the key to success that becomes approachable only after viewing the problem out of the box. Ladurner et al. bring back the opinion of the unification of the RC, the quality of which should be clearly defined, assessed, and supervised (4). On the contrary, personal preferences play here a major role in making the procedure eminence-based in lieu of evidence-based (4). As a result, local modifications of the RC exist, and among them, there is one part of the possible impact on cancer-related survival, i.e., the extent of lymphadenectomy (35). In the current study, we presented an altered version of the original RC-pentafecta, in which the number of LNs was lowered from 16 to 10 along with some other authors’ suggestions as described above. According to Herr et al., a mean of 10–14 nodes should be easy to achieve by an experienced urologist performing RC on a regular basis (36). A paper by Froehner et al. showed that although there was a clear association between LN count and OS after RC (≥21 vs. <10 LNs: 10-year rates: 59% vs. 32%, respectively; hazard ratio (HR) = 0.63; 95% CI 0.46–0.87; p = 0.0056), there was no detectable benefit from the higher number of resected LNs in terms of bladder cancer mortality (p-values ranging between 0.40 and 0.93) (37). Additionally, a prospective randomized controlled trial showed that extended lymphadenectomy does not have the superiority over limited one with regard to RFS (5-year RFS 65% vs. 59%; HR = 0.84; p = 0.36) and CSS (5-year CSS 76% vs. 65%; HR = 0.70; p = 0.10) (38). Moreover, Leminski et al. proved a range of 10–15 LNs to have prognostic significance, while greater numbers of removed nodes resulted in an increase of OS up to 14% (24). According to Koppie et al. (25), removing 10 LNs may represent a thorough LN cleanout from a limited LN template, or a relatively incomplete dissection of LNs from an extended LN template, but found no support for a concept of a minimum number of LNs sufficient for optimizing bladder cancer outcomes. Finally, the sole number of resected LNs is not only a prerequisite of the accurate lymphadenectomy, but the same technique implementation may also result in different numbers removed in the specimen (4).

The main reason for the promotion of pentafecta as a tool for surgical quality assessment is the hypothesis that it can act prognostic factor of oncological outcomes. In our cohort, CSS was greatly affected in the individuals who failed to attain the pentafecta. Laymon et al. reported the discrepancy between pentafecta failers and achievers in 5-year RFS reaching 20% (62.5% vs. 81.7%; p < 0.0001) (34). Then, Oh et al. that studied the Korean Robot-Assisted Radical Cystectomy Study Group observed significantly greater OS and CSS in the group that achieved pentafecta and reported a 48% decrease in the overall mortality in those patients (23). As a consequence, the quality of RC defined by pentafecta achievement may serve as an additional clinical prognosticator, when stepwise management of the disease is planned to increase poor results of the unimodal approach (39, 40).

To determine the chance of pentafecta achievement, we focused on the search for its strong predictors, which were proved in multivariate analyses to be as follows: the surgical approach, the implemented method of UD, histological type of cancer, and advancement of the disease together with elevated serum creatinine level. The above factors might guide clinical decisions in preoperative workup to plan the most optimal surgery (e.g., decisions on UD type). Some authors formulated the hypothesis that surgeons favor ileal conduit, as they associate it with a low likelihood of UD-related complications and good quality of life of patients (41, 42). In the current cohort, both open and laparoscopic approaches were incorporated so the UDs were conducted extracorporeally, and ileal conduit or neobladder was selected in nearly 50% of cases. Interestingly, the data from the paper on robot-assisted technique did not prove the UD type to be a game-changer in the cohort of patients undergoing RC as far as pentafecta attainment is concerned (23). Furthermore, a recent randomized controlled trial comparing robot-assisted and open approaches in terms of quality of life revealed the superiority of the latter in terms of urinary symptoms and problems (43). Clinical and pathological staging regarding LNs appeared not to be the factor determining the chance of achieving pentafecta in the multivariable model. This might reflect the fact that other factors were of more pronounced prognostic value (e.g., surgery modality, pT category, and UD type). Interestingly, other papers also did not directly show the independent prognostic significance of LN staging on trifecta (15) or pentafecta (22). In the recent paper by Oh et al., pentafecta failers were the predominant group, reaching over 71% (23). One should take into consideration similar basic characteristics of Oh’s studied group, i.e., mainly males with a mean age of nearly 65 years, and over 53% with ileal conduit performed. In the recent meta-analysis, though, the robot-assisted approach was superior to open surgery in terms of blood loss and the need for transfusion but not LOH, complications, or mortality (44).

We also found the predictors of cancer recurrence post cystectomy to be as follows: pT staging, presence of PSMs, blood transfusions, number of resected LNs, and presence of positive LNs. Other authors found that negative predictors of pentafecta in multivariate analysis were ASA ≥ 3, BMI ≥ 35, perioperative blood transfusion, and type of UD (ileal conduit) (34). A recent meta-analysis on 7,080 patients revealed the association between the elevated risks of cancer-specific mortality and recurrence in individuals that were transfused (45). A special impact of blood transfusion on CSS after RC was observed regardless of the tumor stage diagnosed (46). It seems that the risk increases along with the number of units transfused (47). As for the postoperative complications, we noted them more frequently in obese individuals and in patients with elevated preoperative creatinine. Then, neither age nor gender was an important predictor, but we enrolled predominantly males with a median of 68 years. On the contrary, Aziz et al. observed that age (median 66) was the independent predictor of tri- and pentafecta but strongly advised to meticulously qualify elderly for the RC and not judge them simply on their birthdate (20). Then, Galette et al. (48) pinpointed that surgeons qualifying for the RC should use geriatric assessment tools rather than chronological age > 75 years, as some authors enrolled even octogenarians (49).

Finally, Baron et al. hypothesized about incorporation into the existing models’ other factors, e.g., LOH, readmission rates, or use of NAC (22). Their utilization might increase the prognostic value of the pentafecta transforming it into hepta- or even octafecta tool. Till now, however, there are no comparisons of the utility of the tri- or pentafecta and even enlarged models in the RC setting that suits them best in the clinical scenario, which should be determined.

We are aware of the inherent limitations of the retrospective nature of our study. The majority of the patients underwent open surgery. We incorporated different centers with surgeons of undetermined experience, using various techniques (including lymphadenectomy), so the impact of hospital volume remains hard to assess. The extent of LN dissection and a way of its submission (either a small or large packet) for pathology assessment was left at the discretion of the surgeon. Pathological assessment of LNs and its number reporting might have differed between centers and pathologists. The median follow-up was 18.5, which is similar to that reported by other authors (20, 21) due to the construction of the study focused on the analysis < 12 months, and long-term data were not available. Using the abovementioned RC-pentafecta definition, all patients with < 12 months of follow-up were not included unless study endpoints (death or cancer recurrence) occurred <12 months. Finally, for additional calculations, other composite outcomes indicating surgical excellence were established, trifecta (achievement of 12 months RFS after RC, no complications related to UD < 12 months, no Clavien–Dindo grade III–V complications at 90 days) and heptafecta (incorporating additional data on duration of hospitalization ≤14 days and no readmissions after RC); but we failed to determine the most optimal set of clinical factors in subanalyses due to the inadequate number of the studied group.

## Conclusions

In conclusion, we found that the pentafecta achievement rate was low in the cohort of muscle-invasive and high-risk non-MIBC patients undergoing RC even in high-volume centers. The complexity of the procedure seems to directly influence the attainment rate, which in turn leads to a great increase in cancer-specific mortality rate among the pentafecta failers. As for the strongest predictors of the pentafecta achievement, we found the surgical approach, type of urinary diversion, histological type of bladder cancer, advanced staging, and increased preoperative serum creatinine to be significant. Further incorporation of the centers into the project might help in the establishment of the direct comparison between different tools, i.e., tri- or pentafecta, or enrich the existing ones with other clinical values.

## Data Availability Statement

The raw data supporting the conclusions of this article will be made available by the authors, without undue reservation.

## Ethics Statement

The studies involving human participants were reviewed and approved by the Ethics committee vote AKBE/133/2021 of the Medical University of Warsaw. The patients/participants provided their written informed consent to participate in this study.

## Author Contributions

Conceptualization: ŁZ and AŚ. Methodology: ŁZ and AŚ. Software: AŚ. Validation: ŁZ, AŚ, and PZ. Formal analysis: ŁZ and AŚ. Investigation: ŁZ, BK, PK, ML, PL, JM, MN, KP, MPo, MPr, TS, and RW. Resources: ŁZ, BK, PK, ML, PL, JM, MN, KP, MPo, MPr, TS, and RW. Data curation: ŁZ and AŚ. Writing: ŁZ and AŚ. Writing and re-editing: ŁZ, AŚ, and PZ. Visualization: ŁZ. Supervision: TD, MR, WR, AW, and PR. Project administration: ŁZ. Funding acquisition: ŁZ. All authors have read and agreed to the published version of the manuscript.

## Funding

This work was supported by the Medical University of Warsaw (Publication Found).

## Conflict of Interest

The authors declare that the research was conducted in the absence of any commercial or financial relationships that could be construed as a potential conflict of interest.

## Publisher’s Note

All claims expressed in this article are solely those of the authors and do not necessarily represent those of their affiliated organizations, or those of the publisher, the editors and the reviewers. Any product that may be evaluated in this article, or claim that may be made by its manufacturer, is not guaranteed or endorsed by the publisher.
