# Supplementary material for: The View Outside of the Box: Reporting Outcomes Following Radical Cystectomy Using Pentafecta From a Multicenter Retrospective Analysis
Source: Front Oncol. 2022 Jan 26;12:841852. doi: 10.3389/fonc.2022.841852 (PMC8828538; doi:10.3389/fonc.2022.841852)
Supplement: Supplementary file 1 [file Table_1.docx]

**Supplementary material**

**Supp. Tab. 1. Factors influencing pentafecta achievement in the subgroup of patients (N=203), who underwent open radical cystectomy with ileal conduit or TU-UCS/UCS as UD type - multivariate analysis.**

|  | OR | 95% CI | P-value |
| --- | --- | --- | --- |
| Ileal conduit vs UCS/TU-UCS (ref) | 4.010 | 1.626-9.887 | 0.0026 |
| Pure urothelial histology vs other (ref) | 0.351 | 0.127-0.969 | 0.0432 |
| pT2-T4 | 0.356 | 0.153-0.825 | 0.0161 |

**Supp. Tab. 2. Factors influencing bladder cancer recurrence in the subgroup of patients (N=203), who underwent radical open cystectomy with ileal conduit or TU-UCS/UCS as UD type - multivariate analysis.**

|  | OR | 95% CI | P-value |
| --- | --- | --- | --- |
| pT3-T4 | 4.796 | 2.337-9.841 | <.0001 |
| Negative surgical margin | 0.345 | 0.123-0.968 | 0.0431 |
| LN count | 0.942 | 0.893-0.993 | 0.0267 |
| Lymph node status |  |  |  |
| pN1-3 vs pN0 (ref) | 2.259 | 1.035-4.933 | 0.0146 |
| pNx vs pN0 (ref) | 4.772 | 1.213-18.77 |  |
